# Phenobarbital Versus Lorazepam for Management of Alcohol Withdrawal Syndrome: A Retrospective Cohort Study

**DOI:** 10.7759/cureus.13282

**Published:** 2021-02-11

**Authors:** Fadi Hawa, Linsey Gilbert, Benjamin Gilbert, Vanessa Hereford, Aya Hawa, Alsadiq Al Hillan, Mark Weiner, Jeremy Albright, Caleb Scheidel, Ola Al-Sous

**Affiliations:** 1 Internal Medicine, St. Joseph Mercy Ann Arbor Hospital, Ann Arbor, USA; 2 Internal Medicine/Palliative Care, St. Joseph Mercy Ann Arbor Hospital, Ann Arbor, USA; 3 Internal Medicine, St. Joseph Mercy Livingston Hospital, Howell, USA; 4 Pharmacy, St. Joseph Mercy Ann Arbor Hospital, Ann Arbor, USA; 5 Internal Medicine/Hepatology, Baylor College of Medicine, Houston, USA; 6 Internal Medicine/Addiction Medicine, St. Joseph Mercy Ann Arbor Hospital, Ann Arbor, USA; 7 Statistics, St. Joseph Mercy Ann Arbor Hospital, Ann Arbor, USA

**Keywords:** alcohol misuse, alcohol withdrawal syndrome, alcohol addiction, phenobarbital, lorazepam, length of hospital stay (los), hospitalized patients

## Abstract

Introduction

Annually, 500,000 episodes of alcohol withdrawal syndrome (AWS) are severe enough to require clinical attention. A symptom-triggered lorazepam regimen remains the standard of care for the management of hospitalized AWS patients. However, phenobarbital has also been shown to be an effective adjunctive therapy for severe AWS, reducing benzodiazepine use in the emergency department (ED) and the intensive care unit (ICU). The purpose of this study is to compare hospital length of stay (LOS) for AWS patients using phenobarbital-based versus lorazepam-based treatment protocols as monotherapy for management of AWS on general medical units.

Methods

This is a retrospective cohort study over a two-year period (March, 2016 to March, 2018), conducted at three hospitals within the St. Joseph Mercy Health System. We included 606 patients with a primary diagnosis of AWS or alcohol intoxication who met our inclusion criteria (543 in the lorazepam cohort and 63 in the phenobarbital cohort). Adjusted comparisons were done using propensity scoring methods. Hospital LOS was set as the primary outcome. Secondary outcomes included all-cause 30-day readmission, alcohol-related 30-day readmission, 30-day ED visits after discharge, and need for ICU transfer during hospital stay.

Results

Patients who received phenobarbital had a statistically significant shorter hospital LOS as compared to patients who received lorazepam (2.8 versus 3.6 days, P < 0.001). Furthermore, the phenobarbital treatment group had statistically significant lower rates of all-cause 30-day readmission (11.11% versus 14.18%, P = 0.020) and 30-day ED visits after discharge (11.11% versus 18.6%, P = 0.015). No statistical significance was detected for alcohol-related 30-day readmission and the need for ICU transfer between the treatment groups.

Conclusion

This study suggests that phenobarbital may be a reasonable alternative to lorazepam in the management of AWS patients admitted to general medical units. Larger scale, well-executed, and adequately powered prospective studies and randomized controlled trials are needed to corroborate these findings.

## Introduction

Alcohol is the most widely available and abused substance in the United States. An estimated 1.2 million hospital admissions are related to alcohol abuse annually, and about 500,000 episodes of withdrawal symptoms are severe enough to require clinical attention annually [1]. Alcohol withdrawal syndrome (AWS) diagnosis is primarily made based on the Diagnostic and Statistical Manual of Mental Disorders (DSM-V) criteria [2].

The Clinical Institute Withdrawal Assessment for Alcohol Scale, Revised (CIWA-AR) is the most widely used scoring tool to guide symptom-triggered therapy [3]. This therapy method is beneficial in individualizing treatments, reducing both treatment duration and the amount of medications used, and is as efficacious as standard fixed-schedule therapy for alcohol withdrawal [3].

The currently accepted treatment regimens have varied but generally include both benzodiazepine (BZD) and non-BZD approaches [4]. Sedative-hypnotic drugs were recommended by the American Society of Addiction Medicine as the primary agents for managing alcohol withdrawal delirium [5]. Current evidence does not strongly indicate that a specific sedative-hypnotic agent is superior to others or that switching from one to another is helpful [5].

Benzodiazepines are most commonly used and recommended by addiction specialists because of their favorable therapeutic/toxic effect index [6]. The effectiveness of BZDs for the management of AWS has been demonstrated across multiple studies both in improving discomfort associated with acute withdrawal, and in decreasing the risk of progression to seizures and alcohol withdrawal delirium [3,6]. However, patients with chronic heavy alcohol use usually acquire tolerance to alcohol and can even develop cross-tolerance to BZDs [7]. Furthermore, BZDs may be associated with additional risks of oversedation, encephalopathy, and agitation in medically hospitalized patients. In addition, BZDs have been associated with an increased risk of rebound withdrawal symptoms and post-treatment drinking in multiple randomized controlled trials conducted in an ambulatory setting for AWS patients [8-12].

Barbiturates, such as phenobarbital have been proven to be clinically safe, cost-effective, and easy-to-use medications and can potentially be considered as alternatives to BZDs [13,14]. They have different binding properties and receptor affinity when compared to BZDs that are believed to result in a reduced cross-tolerance between phenobarbital and alcohol [15,16]. Moreover, commonly used doses of phenobarbital for AWS have not been associated with clinically significant sedation [17].

Recent literature supports the similarity between phenobarbital and BZDs with regard to tolerability and effectiveness in the treatment of AWS [17,18]. Despite these trends, there is only modest evidence comparing hospital length of stay (LOS) as a primary outcome between phenobarbital and BDZs in management of AWS patients admitted to general medical units in acute-care hospital setting [17-21].

Within our health system (St. Joseph Mercy Health System) which is comprised of five Michigan hospitals, there is a CIWA-AR dose-based driven protocol in place that allows physicians to choose different pharmaceutical options and routes of administration for management of AWS. The two existing options within this protocol are lorazepam and phenobarbital, orally, intramuscularly or intravenously. The choice of medication is mainly driven by the treating physician’s preference. In general medical units, different trends towards which medication used have been noted among these hospitals within the same health system. However, they all share the practice of using phenobarbital if a patient is transferred to the intensive care unit (ICU). Given the observed internal variance within our health system and lack of quality data regarding AWS management, we compared the two protocols employed across three of our health system hospitals (Hospital A, Hospital B, and Hospital C). 

Our study aimed to compare hospital LOS for AWS patients using phenobarbital-based versus lorazepam-based treatment protocols as monotherapy for the management of AWS on general medical units. We hypothesized no significant difference in hospital LOS between phenobarbital and lorazepam. This article was previously presented as a meeting abstract at the 2020 AAAP Annual Scientific Meeting on December 8, 2019 [22]. 

## Materials and methods

Study design

This is a retrospective cohort study for patients who were admitted to general medical units of three hospitals (Hospital A, Hospital B, and Hospital C) within the same health system (St. Joseph Mercy Health System) with a primary diagnosis of alcohol intoxication or AWS over a two-year period from March, 2016 to March, 2018. Hospital A is a 537-bed tertiary teaching hospital with a case-mix index-adjusted length of stay (CMI-ALOS) of 3.06, Hospital B is a 136-bed teaching hospital with CMI-ALOS of 2.57, and Hospital C is a 133-bed teaching hospital with CMI-ALOS of 2.49. CMI-ALOS is defined as the ratio of the number of days of hospital care that were utilized to care for patients adjusted for the documented severity of the illnesses [23].

Hospital LOS was defined as the primary outcome for the study. Secondary outcomes included, need for ICU transfer, all-cause 30-day readmission rate, alcohol-related 30-day readmission rate (both inpatient and observation statuses were included as a readmission event), and 30-day emergency department (ED) visits after discharge.

This study was intended to be a pilot observational study based on the small cohort of patients, given the low prevalence of alcohol withdrawal in the inpatient population and the need for a large sample size to power a non-inferiority study.

Inclusion and exclusion criteria 

Eligible patients were adults aged 18-100 years without sex discrimination, who were admitted for alcohol intoxication or withdrawal to any of the above-defined facilities. Patients were excluded if they were admitted directly to ICU, pregnant women, prisoners, and patients who were transferred from an outside facility, or received an addiction medicine specialist consultation.

Variables

Data were obtained retrospectively through accessing the electronic medical records post-hospitalization. Data was abstracted both electronically and manually. Double data abstraction was used for manual abstractors until inter-rater reliability was achieved. Data was then reviewed using the inclusion and exclusion criteria. Collected variables included general demographics (gender, age, and ethnicity), major medical comorbidities (seizure disorder, coronary artery disease, hypertension, diabetes mellitus, chronic kidney disease, chronic obstructive pulmonary disease, and liver disease), AWS-related variables (initial CIWA score, and maximum CIWA score), outcome-related variables (hospital LOS, need for ICU transfer, alcohol-related 30-day readmission rate, all-cause 30-day readmission rate, and 30-day ED visits after discharge). Further variables that could have potentially influenced treatment decision or outcome were collected, which included presence or absence of urine drug screen, seizure incidence during hospitalization, depression, anxiety, schizophrenia, schizoaffective disorder, bipolar disorder, discharge to inpatient psychiatry unit, and concomitant use of gabapentin.

Statistical analysis

Unadjusted comparisons for all covariates and study outcomes by treatment type were assessed using chi-square or Fisher exact tests for categorical data and t-tests or Mann-Whitney tests for interval-level variables. Adjusted comparisons were conducted using propensity score methods. Specifically, propensity scores (the probability of receiving each treatment) were estimated based on a model in which all covariates are entered as predictors. The propensity scores were then converted to weights, and the tests were repeated using the propensity score-weighted data. Propensity score weights were preferable to matching for determining adjusted treatment effects given that no observations were discarded. All analyses were completed using R Version 3.6.0 [24], assuming a significance level of 0.05. 

Ethical consideration

The study was approved by the St. Joseph Mercy Health System Institutional Review Board. Informed consent was waived given the retrospective nature of the study.

## Results

**Figure 1:**
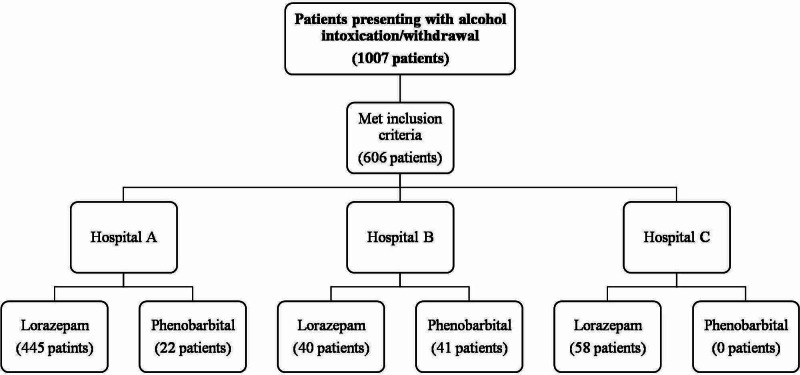
Flow diagram detailing the number of patients who received each treatment per hospital.

A total of 1,007 charts for patients admitted with a diagnosis of alcohol intoxication or withdrawal during the time period March, 2016 to March, 2018 were reviewed. After applying the exclusion criteria, 606 patients were included in the study (543 in the lorazepam cohort and 63 in the phenobarbital cohort) (Figure 1). 

Table 1 demonstrates baseline demographics and clinical characteristics among treatment groups. The treatment groups are balanced by all confounders except for initial CIWA score and presence of urine drug screen before propensity score weighing. After weighing the treatment groups by propensity scores, the groups are balanced by all confounders except for chronic kidney disease (CKD), which is likely due to the fact that the phenobarbital treatment group had zero patients with CKD (Table 1).

**Table 1:** Patients' baseline demographics and clinical characteristics - all hospitals (n = 606). Propensity score weighing is used to balance treatment groups by all confounders. PS: propensity score; CIWA: Clinical Institute Withdrawal Assessment.

Variable	Label	Lorazepam (n = 543)	Phenobarbital (n = 63)	Unadjusted P-value	PS weighted P-value
Categorical variables, n (%)					
Sex				0.546	0.919
	Female	173 (31.86%)	23 (36.51%)		
	Male	370 (68.14%)	40 (63.49%)		
Ethnicity				>0.999	0.622
	Black	38 (7%)	4 (6.35%)		
	White	502 (92.45%)	59 (93.65%)		
	Declined	3 (0.55%)	0 (0%)		
Hospital				<0.001	<0.001
	Hospital A	445 (81.95%)	22 (34.92%)		
	Hospital B	40 (7.37%)	41 (65.08%)		
	Hospital C	58 (10.68%)	0 (0%)		
Anxiety		216 (39.78%)	27 (42.86%)	0.737	0.767
Bipolar disorder		75 (13.81%)	9 (14.29%)	>0.999	0.701
Coronary artery disease		22 (4.05%)	2 (3.17%)	>0.999	0.67
Depression		201 (37.02%)	23 (36.51%)	>0.999	0.541
Diabetes mellitus		35 (6.45%)	2 (3.17%)	0.412	0.493
Hypertension		241 (44.38%)	27 (42.86%)	0.923	0.827
Liver disease		133 (24.49%)	18 (28.57%)	0.579	0.698
Schizoaffective disorder		7 (1.29%)	1 (1.59%)	0.587	0.828
Schizophrenia		13 (2.39%)	2 (3.17%)	0.663	0.867
Chronic kidney disease		11 (2.03%)	0 (0%)	0.615	0.009
Chronic obstructive pulmonary disease		43 (7.92%)	3 (4.76%)	0.461	0.302
Seizure incidence during hospitalization		10 (1.86%)	1 (1.64%)	>0.999	0.991
Urine drug screen		339 (62.43%)	51 (80.95%)	0.006	0.104
Use of gabapentin		159 (29.28%)	18 (28.57%)	>0.999	0.962
Discharge to inpatient psychiatry unit		37 (6.81%)	4 (6.35%)	>0.999	0.644
Blood alcohol level				0.865	0.906
	<10	133 (26.55%)	15 (25.86%)		
	10-100	72 (14.37%)	8 (13.79%)		
	101-200	63 (12.57%)	6 (10.34%)		
	201-300	87 (17.37%)	14 (24.14%)		
	301-400	95 (18.96%)	9 (15.52%)		
	>400	51 (10.18%)	6 (10.34%)		
Continuous variables, mean (SD)					
Age		47.178 (12.742)	45.568 (13.695)	0.377	0.666
Initial CIWA score		9.046 (5.87)	11.016 (7.54)	0.049	0.859
Max CIWA		15.904 (7.073)	16.286 (6.566)	0.666	0.29

Comparing the two treatment groups across the three hospitals and after adjusting for baseline demographic and clinical variables, patients in the phenobarbital treatment group had a statistically significant shorter mean LOS when compared to patients in the lorazepam treatment group (2.8 versus 3.6 days, P < 0.001). In addition, patients in the phenobarbital treatment group had a statistically significant lower all-cause 30-day readmission rate (11.11% versus 14.18%, P = 0.020) and 30-day ED visits after discharge (11.11% versus 18.6%, P = 0.015). There was no statistical significance detected for alcohol-related 30-day readmission and need for ICU transfer between the treatment groups (Table 2). 

**Table 2:** Primary and secondary outcomes – all hospitals (n = 606). Propensity score weighing is used to balance treatment groups by all confounders. ED: emergency department; ICU: intensive care unit; LOS: length of stay; PS: propensity score.

Primary and secondary outcomes	Lorazepam (n = 543)	Phenobarbital (n = 63)	Unadjusted P-value	PS weighted P-value
Categorical outcomes, N (%)				
Transferred to ICU	38 (7.04%)	2 (3.23%)	0.416	0.114
All-cause 30-day readmission	77 (14.18%)	7 (11.11%)	0.635	0.020
Alcohol-related 30-day readmission	65 (11.97%)	6 (9.52%)	0.715	0.045
ED visit within 30 days of discharge	101 (18.6%)	7 (11.11%)	0.195	0.015
Continuous outcomes, mean (SD)				
LOS (days)	3.664 (2.324)	2.805 (1.255)	<0.001	<0.001

Hospital B was noted to have a relatively equal patient distribution among treatment groups. Accordingly, a subgroup analysis for Hospital B was performed. The treatment groups are balanced by all confounders except for anxiety before propensity score weighing. After weighing the treatment groups by propensity scores, the groups are balanced by all confounders (Table 3).

**Table 3:** Baseline demographics and clinical characteristics - Hospital B (n = 81). Propensity score weighing is used to balance treatment groups by all confounders. CIWA: Clinical Institute Withdrawal Assessment; PS: propensity score.

Variable	Label	Lorazepam (n = 40)	Phenobarbital (n = 41)	Unadjusted P-value	PS weighted P-value
Categorical variables, n (%)					
Sex				0.753	0.693
	Female	16 (40%)	14 (34.15%)		
	Male	24 (60%)	27 (65.85%)		
Ethnicity				-	-
	White	40 (100%)	41 (100%)		
Anxiety		11 (27.5%)	22 (53.66%)	0.03	0.41
Bipolar disorder		1 (2.5%)	5 (12.2%)	0.201	0.085
Coronary artery disease		0 (0%)	1 (2.44%)	>0.999	0.327
Depression		13 (32.5%)	15 (36.59%)	0.878	0.794
Diabetes mellitus		1 (2.5%)	0 (0%)	0.494	0.293
Hypertension		14 (35%)	16 (39.02%)	0.885	0.712
Liver disease		9 (22.5%)	13 (31.71%)	0.495	0.533
Schizoaffective disorder		0 (0.00%)	0 (0.00%)	-	-
Schizophrenia		0 (0%)	1 (2.44%)	>0.999	0.341
Chronic kidney disease		0 (0.00%)	0 (0.00%)	-	-
Chronic obstructive pulmonary disease		10 (25%)	3 (7.32%)	0.062	0.109
Seizure incidence during hospitalization		0 (0%)	1 (2.56%)	0.494	0.328
Urine drug screen		32 (80%)	35 (85.37%)	0.73	0.381
Use of gabapentin		18 (45%)	12 (29.27%)	0.217	0.229
Discharge to inpatient psychiatry unit		3 (7.5%)	1 (2.44%)	0.359	0.193
Blood alcohol level				0.746	0.996
	<10	8 (20.51%)	9 (24.32%)		
	10-100	8 (20.51%)	5 (13.51%)		
	101-200	4 (10.26%)	3 (8.11%)		
	201-300	6 (15.38%)	10 (27.03%)		
	301-400	7 (17.95%)	7 (18.92%)		
	>400	6 (15.38%)	3 (8.11%)		
Continuous variables, mean (SD)					
Age		44.154 (11.911)	43.723 (10.914)	0.866	0.832
Initial CIWA score		11.625 (7.11)	12.61 (8.053)	0.561	0.819
Max CIWA		17.6 (6.484)	16.878 (6.29)	0.613	0.615

Patients receiving either lorazepam or phenobarbital at Hospital B were then compared for primary and secondary outcomes after adjusting for baseline demographic and clinical variables. There was no statistical significance detected between treatment groups in regards to hospital LOS, need for ICU transfer or 30-day readmission rate. However, the phenobarbital treatment group had a statistically significant lower rate for 30-day ED visits after discharge when compared to patients in the lorazepam treatment group (14.63% versus 35%, P = 0.017) (Table 4).

**Table 4:** Primary and secondary outcomes – Hospital B (n = 81). Propensity score weighing is used to balance treatment groups by all confounders. ED: emergency department; ICU: intensive care unit; LOS: length of stay; PS: propensity score.

Sub-group analysis outcome (Hospital B)	Lorazepam (n = 40)	Phenobarbital (n = 41)	Unadjusted P-value	PS weighted P-value
Categorical outcomes, n (%)				
Transferred to ICU	3 (7.5%)	1 (2.5%)	0.615	0.267
All-cause 30-day readmission	5 (12.5%)	2 (4.88%)	0.264	0.188
Alcohol-related 30-day readmission	4 (10%)	2 (4.88%)	0.432	0.353
ED visit within 30 days of discharge	14 (35%)	6 (14.63%)	0.062	0.017
Continuous outcomes, mean (SD)				
LOS (Days)	2.999 (1.103)	2.689 (1.138)	0.218	0.200

## Discussion

This is a pilot retrospective cohort study which compared the use of phenobarbital and lorazepam in the management of patients with AWS admitted to general medical units. The study included patients from three hospitals within the same health system. These hospitals have relatively similar CMI-ALOS and also share very similar workflow standards and treatment protocols. In this study, phenobarbital had a statistically significant shorter hospital LOS when compared to lorazepam. Furthermore, the phenobarbital treatment group had a statistically significant lower all-cause 30-day readmission rates and 30-day ED visits after discharge. Other secondary outcomes including alcohol-related 30-day readmission rate and need for ICU transfer showed no statistically significant difference.

A subgroup analysis for Hospital B was conducted given a relatively equal patient distribution across treatment groups (41 patients in the phenobarbital cohort and 40 patients in the lorazepam cohort). This has revealed no statistically significant difference in hospital LOS between treatment groups. Secondary outcomes were only statistically significant for a lower rate of ED visits within 30 days of discharge for phenobarbital when compared to lorazepam.

The propensity scoring method was done to ensure adjusted comparisons between the treatment groups especially for factors that may play a role in the management of AWS. Psychiatric disorders, inpatient psychiatry disposition, substance use disorders, or concomitant use of gabapentin are factors that might have either influenced the treatment decision or confounded the studied outcomes. Although the treatment groups were mostly balanced prior to propensity score weighing, weights were still used in the analysis to ensure comparable treatment groups based on these factors.

Our study results are consistent with the available literature and add to the growing body of evidence that phenobarbital may be a potentially feasible alternative to lorazepam in management of AWS. To our knowledge, this is the first study comparing hospital LOS as a primary outcome between lorazepam and phenobarbital in the treatment of AWS patients admitted to general medical units. 

The safety and utility of phenobarbital in management of AWS as a monotherapy or as an adjunct to BZD, has been evaluated across multiple clinical settings [19-21,25-27]. Mo et al. reviewed a group of studies including 4 prospective controlled and 3 retrospective clinical trials to assess the efficacy and safety of barbiturates with or without BZDs versus BZDs for the treatment of AWS in the acute setting [17]. None of the studies reviewed demonstrated the inferiority of barbiturates to BZD in the management of AWS. Furthermore, overall safety profiles of barbiturates were comparable to those of BZDs across all studies included. Hammond et al., evaluated patient outcomes associated with phenobarbital use with or without BZDs for AWS by reviewing four controlled trials and five observational studies [18]. These authors concluded that phenobarbital may have a role in AWS treatment alongside BZDs or as monotherapy. Furthermore, patients with severe AWS who received phenobarbital required less escalation of their care, and those with mild to moderate AWS spent less time in the ED and did not require further care following discharge. These findings were suggestive of similar or even improved outcomes with phenobarbital when compared to alternative therapies, including BZDs. Unfortunately, both of these systematic reviews lacked high-quality evidence and contained a high degree of heterogeneity of the included studies, preventing the performance of a meta-analysis.

Two randomized controlled trials conducted in an ED setting evaluated the outcomes of phenobarbital for AWS management. The first is a placebo-controlled trial that studied the initial level of hospital admission (ICU versus telemetry versus floor wards) [19]. The trial showed that patients receiving a single dose of intravenous phenobarbital had a decreased ICU admission rate (phenobarbital versus placebo, 8% versus 25% with 95% confidence interval [4%-32%]). No statistical differences were noted in admission neither to telemetry or floor wards nor in median ICU or total hospital LOS. Furthermore, there was no difference in the incidence of adverse outcomes between phenobarbital and placebo. The second trial compared intravenous phenobarbital to intravenous lorazepam in the treatment of acute AWS with regard to efficacy to improve symptoms during ED visits and at 48-hour reassessment as the primary outcome [20]. There was no statistical difference between phenobarbital and lorazepam in baseline CIWA scores (P = 0.3), at ED discharge (P= 0.4), or at 48-hour re-evaluation (P = 0.7). Therefore, phenobarbital was found to be as effective as lorazepam in the treatment of acute AWS.

Nisavic et al. conducted a retrospective review to evaluate the development of alcohol withdrawal-related complications following the initiation of treatment with either phenobarbital or BZD [21]. No statistical significance was detected between the two treatment protocols from a primary clinical outcome standpoint including alcohol-related seizure activity, hallucination, or delirium. Furthermore, no difference was detected in any of the study's secondary outcomes including hospital LOS, ICU admission rate and LOS, medication-related adverse events, or discharge against medical advice. Thus, offering further evidence that phenobarbital, like BZDs, appears to be a well-tolerated and effective treatment for AWS.

The main limitation of our study is that the population comes from three hospitals with a relatively unbalanced number of patients in each treatment group (543 in the lorazepam cohort and 63 in the phenobarbital cohort). This creates an inherited bias that cannot be controlled for as it is related to the treating physician's preference to a specific treatment protocol (phenobarbital versus lorazepam) in addition to the individual hospital workflow. However, all three hospitals share relatively comparable CMI-ALOS and are part of the same health system which probably reduces the weight of this limitation. For that reason, the subgroup analysis for Hospital B was performed given the relatively equal patient distribution among treatment groups, which showed no significant difference in hospital LOS between phenobarbital and lorazepam. Nonetheless, these results continue to be in concordance with the available literature suggesting that phenobarbital may be a reasonable alternative to BZDs. Furthermore, there are limits to the study's generalizability as the majority of patients included were white, which is reflective of the three hospitals' local community demographics. Other limitations to the study include its retrospective nature, lack of randomization, and lack of knowledge whether patients were readmitted to other health systems within 30 days after discharge.

## Conclusions

In conclusion, this pilot retrospective cohort study suggests that phenobarbital may be a reasonable alternative to lorazepam in management of AWS patients admitted to general medical units. When taking into account the BZDs-related adverse events including oversedation, encephalopathy, agitation, and increased risk of rebound withdrawal symptoms, phenobarbital may represent a reasonable alternative with a potential for improved outcomes. Larger scale, well-executed, and adequately powered prospective studies and randomized controlled trials are needed to provide conclusive evidence to support the non-inferiority of phenobarbital to BZDs as a treatment option for AWS.
